# Autophagy-targeted intervention synergized with pegaspargase: an innovative therapeutic paradigm for pancreatic cancer

**DOI:** 10.1186/s12935-025-04074-5

**Published:** 2025-11-28

**Authors:** Dandan Zhou, Zhe Ding, Tongjin Yin, Mei Huang, Xuyao Zhang, Honggang Cao, Chunbin Wang

**Affiliations:** 1 Department of Oncology, the Yancheng School of Clinical Medicine of Nanjing Medical University, Yancheng Third People’s Hospital, Yancheng, Jiangsu P.R. China; 2 Department of Pharmacy, the Yancheng School of Clinical Medicine of Nanjing Medical University, Yancheng Third People’s Hospital, Yancheng, Jiangsu P.R. China; 3 Department of Paediatrics, the Yancheng School of Clinical Medicine of Nanjing Medical University, Yancheng Third People’s Hospital, Yancheng, Jiangsu P.R. China; 4 Department of Biological Medicines, School of Pharmaceutical Sciences, Shanghai Engineering Research Center of Immunotherapeutics, Fudan University, Shanghai, P.R. China

**Keywords:** Pegaspargase, Pancreatic cancer, Autophagy, Apoptosis, Combination therapy

## Abstract

Pegaspargase is a pegylated asparaginase formulation used in hematological malignancies. However, its application in solid tumors is greatly limited due to inherent drug resistance nature of solid tumors. Poor prognosis and lack of effective treatment options for pancreatic cancer urgently necessitate the exploration of novel therapeutic approaches. In the present study, we analyzed pegaspargase’s cytotoxicity on pancreatic cancer cells (SU.86.86 and BxPC-3) in vitro. Then we evaluated the level of autophagy and related pathway proteins during treatment. Finally, we aimed to evaluate the effect of autophagy modulator on pegaspargase efficacy both in vitro and in vivo. The results revealed that pegaspargase (4 IU/mL) decreased the viability of SU.86.86 and BxPC-3 cells and induced apoptosis rates of 28.7% and 16.9%, respectively. Pegaspargase activated autophagy and downregulated Akt/mTOR path-way. Furthermore, autophagy inhibitors increased the cytotoxicity of pegaspargase (4 IU/mL) on SU.86.86 and BxPC-3 cell lines and induced apoptosis rates of 39.9% and 21.9%, respectively. In vivo evaluation confirmed that autophagy inhibition enhanced the antitumor efficacy of pegaspargase. In conclusion, our research demonstrated that combining pegaspargase with autophagy inhibition is a promising approach in pancreatic cancer clinical treatment.

## Introduction

Pancreatic cancer is ranked as the sixth leading cause of cancer-related deaths worldwide [1]. Since pancreatic cancer lacks of typical symptoms in early stages, patients with pancreatic cancer are usually identified at a point where it is either unresectable or already metastasized [2]. New targeted and immunotherapy has significantly improved the survival benefits in other types of tumors. Unfortunately, pancreatic cancer still lacks of these benefits [3]. According to the latest data, only 12% of patients with pancreatic cancer could survive for five more years after being diagnosed [4]. Consequently, the dismal prognosis and lack of available treatment alternatives make the search for innovative therapeutic approaches for pancreatic cancer necessary.

Asparaginase is a bacteria-derived enzyme which induces apoptosis by interrupting the DNA, RNA and protein synthesis processes through the breakdown of asparagine [5]. Unlike normal cells, the majority of tumor cells are unable to synthesize asparagine autologically because of the deficiency of asparagine synthetase (ASNS), rendering them sensitive to asparagine depletion [6]. Based on this property, asparaginase is currently a classic drug used in leukemia [7]. Unfortunately, the clinical use of asparaginase in solid tumors still has many challenges. The primary limitation comes from the inherent drug resistance mechanisms of solid tumors further. Studies have revealed that ASNS expression is higher in some solid tumors compared to acute lymphoblastic leukemia (ALL) and this enzyme confers the tumor cells with increased adaptability to asparagine depletiona [8, 9]. The complex microenvironment of solid tumors also plays an important role in drug resistance. Specifically, characteristics of the solid tumor microenvironment, such as high interstitial pressure and hypoxic conditions, bring great challenges to drug entry [10].In addition, stromal cells in the microenvironment may maintain the survival of tumor cells by constructing metabolic compensation networks [11, 12]. Facing the above challenges, it is necessary to optimize the current therapeutic strategies. Pegaspargase, one of the commonly used asparaginase preparations, is characterized by its covalent conjugation with polyethylene glycol (PEG). Compared with the wild enzyme, pegylated asparaginase shows prolonged half-life and decreased immunogenicity, and the incidence of asparaginase antibody is also decreased [13]. Its improved pharmacokinetic profile may possess potential advantages in the application to solid tumors. we hypothesize that screening patient populations with low ASNS expression and developing combination therapies that sensitize patients to pegaspargase could break through existing bottlenecks, effectively reverse tumor drug resistance, and significantly advance the application of asparaginase in treating solid tumors.

Autophagy is essential for sustaining cellular homeostasis through degrading and recycling excess or damaged proteins and organelles [14]. Based on the sensitivity of cancer treatment to autophagy inhibition, the multifunctional roles of autophagy can be classified into four main types: (i) non-protective: not significantly associated with cell fate and not influencing treatment sensitivity; (ii) cytostatic: inhibits tumor growth by suppressing the cell proliferation cycle (e.g., inducing senescence) but not directly killing cells; (iii) cytoprotective: a key survival program of tumor cells and a major cause of drug resistance; (iv) cytotoxic: excessively activated autophagy can directly induce tumor cell death [15–19]. Among these, the cytoprotective role is particularly prominent in cancer therapy, as it enables cancer cell survival under various stress conditions. Antitumor agents, including Nectin-4-MMAE, SIRPα-Fc fusion protein, T-DM1, and arginase activate this protective mechanism, whereas blocking autophagy significantly enhances the sensitivity of aforementioned drugs [20–23]. There also exists a close relationship between asparaginase and autophagy. For instance, Song P et al. have shown that asparaginase triggers chronic myelogenous leukemia (CML) to undergo pro-survival autophagy, and a therapeutic strategy combining it with autophagy suppression enhanced its antitumor efficacy [24]. Comparable results have been observed across a spectrum of other cancer types, including ALL [25], precursor B-cell acute lymphocytic leukemia [26], lung adenocarcinoma [27], and glioblastoma [28]. However, the impact of autophagy on pancreatic cancer under asparaginase induction has not been confirmed to date.

In this study, we first explored the potential antitumor effects and mechanisms of pegaspargase on pancreatic cancer cells in vivo and in vitro. We then analyzed the significance of autophagy in the progression of pancreatic cancer cells in response to pegaspargase treatment. Finally, we combined autophagy inhibitors with pegaspargase and evaluated their synergistic antitumor effects, aiming to explore the feasibility and potential of this combined therapy as a treatment strategy for pancreatic cancer.

## Materials and methods

### Reagents and antibodies

The asparaginase formulation used in this study was pegaspargase injection, purchased from Jiangsu Hengrui Medicine Co., Ltd. (Lianyungang, China). The ASNS, PARP1, mTOR, Akt, p70S6K, 4EBP1 monoclonal antibody (mouse-derived) and LC3 I/II polyclonal antibody (rabbit-derived) were obtained from Proteintech Group, Inc. (Wuhan, China). Unless specifically noted, all other primary antibodies used are rabbit-derived monoclonal antibodies provided by Cell Signaling Technology (CST) (Danvers, USA). These encompass antibodies against β-actin, cleaved caspase-3 (Asp175) (5A1E), phospho-mTOR (Ser2448) (D9C2) XP^®^, phospho-Akt (Ser473) (D9E) XP^®^, phospho-p70S6 kinase (Thr421/Ser424) and phospho-4EBP1 (Thr37/46) (236B4). The corresponding secondary antibodies for the aforementioned primary antibodies were also obtained from CST, USA.

### Cell culture

The human pancreatic cancer cell lines SU.86.86 and BxPC-3, along with the K562 cell line derived from CML, were both acquired from the Shanghai Branch of the Cell Bank, Chinese Academy of Sciences. All cells were cultured in RPMI-1640-based complete media, with high-quality fetal bovine serum (ExCell Bio, Suzhou, China) and antibiotics (Servicebio, Hubei, China) added at concentrations of 10% and 1%, respectively. The culture conditions included a temperature of 37 °C, a gaseous phase containing 5% CO_2_, and an appropriate humidity environment. Routine screening for cellular contaminants, including mycoplasma, was conducted to ensure the accuracy and reproducibility of experimental results.

### Cell viability assay

Based on different experimental objectives, the cells in the 96-well plate were co-incubated under specified conditions for 24, 48, or 72 h. Following this incubation period, the supernatant was carefully aspirated and MTT solution (Meilunbio, Dalian, China; stored under light-protected conditions) was added for a 4-hour incubation in each well. Following this, DMSO was added to thoroughly dissolve the formazan precipitates. Subsequently, an absorbance measurement at 570 nm was conducted utilizing a microplate reader.

### Apoptosis assay

Apoptosis was identified with the Annexin V-FITC/PI Apoptosis Detection Kit, which was obtained from Meilunbio (Dalian, China). Pegaspargase was given to pancreatic cancer cells, either with or without chloroquine (CQ) (Sigma-Aldrich, St. Louis, USA), followed by cell collection, washing, and resuspension in an appropriate volume of binding buffer. Subsequently, the cells were stained according to the operational guidelines. The corresponding compensation experiments were conducted to improve the accuracy of the data. A Beckman flow cytometer was used for the final data analysis.

### Transmission electron microscopy

SU.86.86 and BxPC-3 cells were incubated together with or without pegaspargase at a concentration of 4 IU/mL for a duration of 24 h. Cells then were harvested using trypsin without EDTA (Meilunbio, Dalian, China) and fixed in an adequate amount of glutaraldehyde solution for storage at 4 °C. The autophagic structures in stained slices of the samples were examined using transmission electron microscopy (TEM), and captured images at 3000x and 8000x magnifications for further analysis.

### Western blot analysis

Different concentrations of pegaspargase were used to incubate the pancreatic cancer cells. The concentrations of all samples were normalized for subsequent experiments using the BCA protein assay kit (Abbikine, Wuhan, China). SDS-PAGE (Servicebio, Wuhan, China) was used to separate protein samples from each lane. After blocking the membranes, they underwent overnight incubation with primary antibodies, subsequently being washed thoroughly and then treated with secondary antibodies. The visualization of targeted bands was achieved using an ultra-high sensitivity ECL working solution (Abbikine, Wuhan, China) in conjunction with fluorescence imaging equipment. The density was calculated by the ImageJ software and the results reflected the relative expression level of each protein.

### Confocal Immunofluorescence

To assess the degree of autophagy in pancreatic cancer cells initiated by pegaspargase, SU.86.86 and BxPC-3 cells received treatment with or without pegaspargase (4 IU/mL) for a duration of 24 h in specialized culture dishes. As a positive control, the autophagy inducer rapamycin (50 nM) was utilized to incubate pancreatic cancer cells. Live cells were then adequately marked with Cyto-ID and Hoechst 33,342 dyes in accordance with the operational guidelines. Autophagosomes selectively labeled were observed using confocal microscopy under dark conditions.

### Drug synergy assay

SU.86.86 and BxPC-3 cells were exposed to various concentration combinations of pegaspargase (0, 1, 2, 4, 8 IU/mL) and chloroquine (0, 1, 2.5, 5, 10 µM) in 96-well plates. After 48 h of incubation, cell viability was measured by MTT assay. The obtained inhibition rate data were then imported into the online analysis tool SynergyFinder (https://synergyfinder.fimm.fi), and the zero interaction potency (ZIP) model was selected to calculate the synergy score for each drug combination. A synergy score ≥ 10 indicates a strong synergistic effect.

### Model of tumor xenograft

To evaluate and compare the antitumor activity of different therapeutic regimens against pancreatic cancer cells in vivo, BALB/c nude mice (male, 6 weeks old) were purchased and housed under standard conditions for the establishment of SU.86.86 xenograft models. The harvested SU.86.86 cells were injected subcutaneously at a standard dose of 1 × 10^7^ cells per mouse. After successful establishment of the animal models, the mice were allocated at random into five groups. Pegaspargase and gemcitabine were administered intravenously, while chloroquine was given via intraperitoneal injection. Throughout the experiment, mouse body weight and tumor volumes were measured and recorded regularly. The tumors and major organs were subsequently resected for histological examination. The animal experiment was carried out in compliance with the guidelines established by the Fudan University School of Pharmacy’s Animal Ethical Committee (2025-01-SY-ZXY-02).

### statistical analysis

In this study, the creation of the graphs was facilitated by Adobe Photoshop and Illustrator. GraphPad Prism 9 and Excel were utilized for data analysis, the results were presented as mean ± SD. Student’s t-test and one-way ANOVA statistical methods were applied to determine the statistical significance between groups. *P* values < 0.05 and < 0.01 were denoted by * and **, respectively.

## Results

### Pegaspargase induced potent cytotoxicity and increased caspase-mediated apoptosis in pancreatic cancer cells

**Fig. 1:**
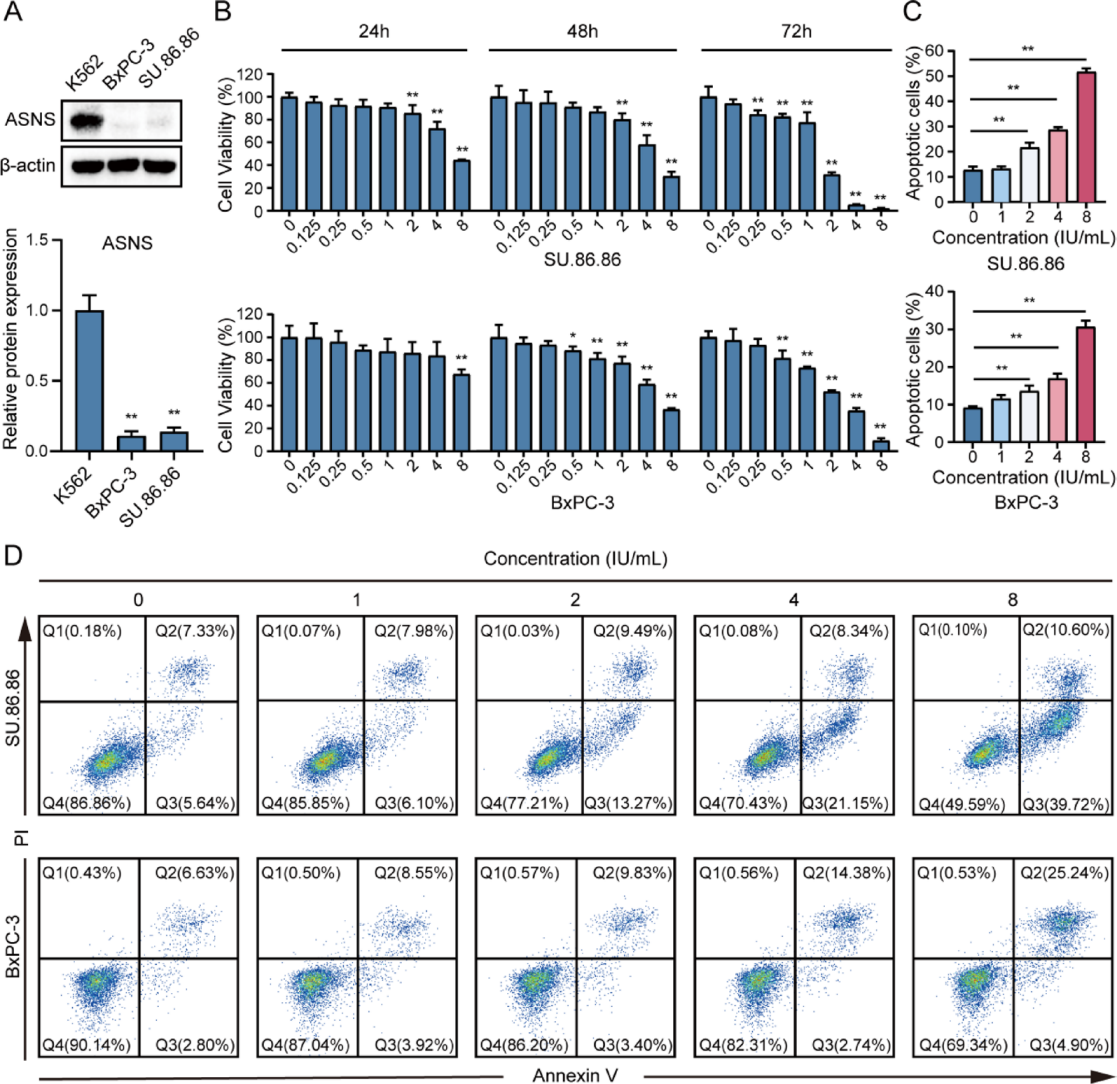
Pegaspargase exerted potent cytotoxicity against SU.86.86 and BxPC-3 cells (**A**) Western blot was performed to assess ASNS expression in SU.86.86 and BxPC-3 cells. The positive control used K562 cells. The densitometric values were estimated utilizing the ImageJ software program. (**B**) The cell viability of pancreatic cancer cells was assessed by MTT assay. (**C**, **D**) Apoptosis in BxPC-3 and SU.86.86 cells was examined and the results were presented in the form of bar graphs. P values < 0.05 and < 0.01 were denoted by * and **, respectively.

**Fig. 2:**
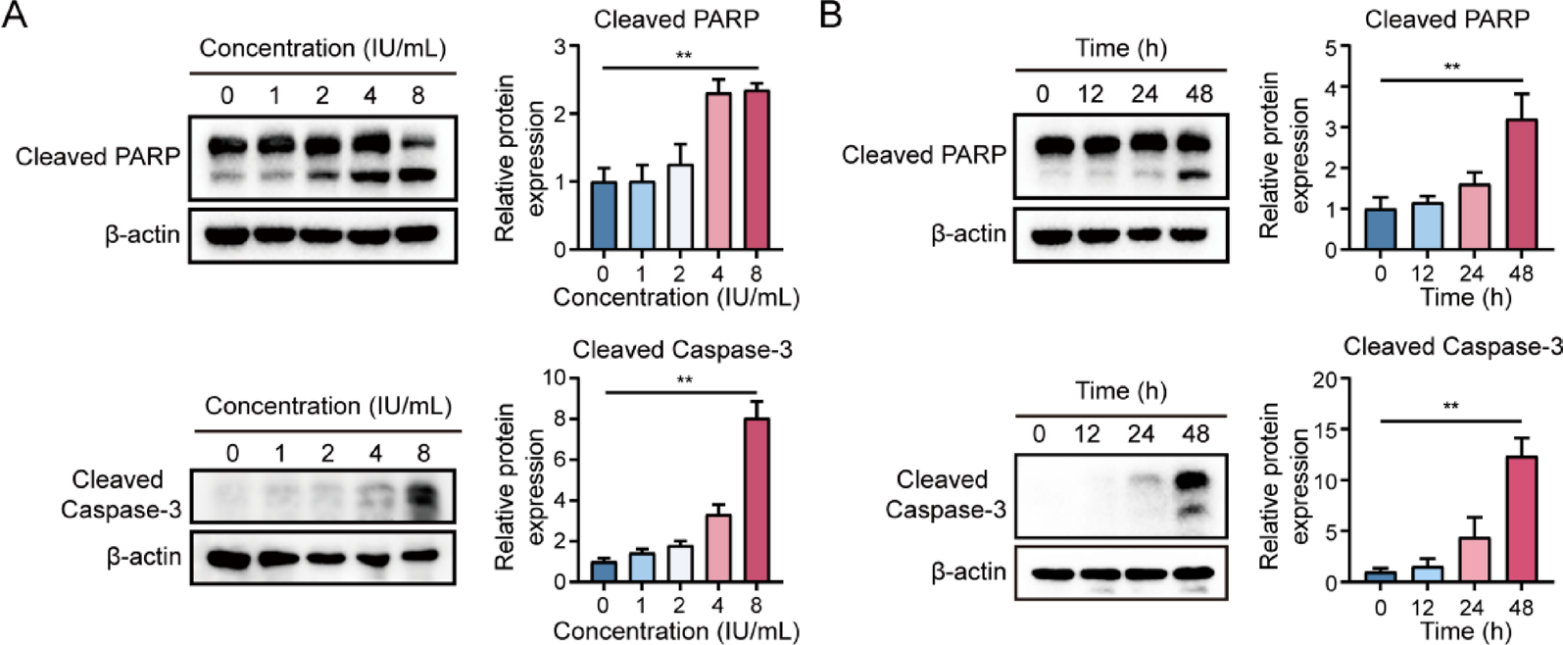
Caspase-3-mediated apoptotic pathways was activated by pegaspargase in pancreatic cancer cells. (**A**) After 48 hours of treatment with pegaspargase, the levels of apoptosis-related proteins (Cleaved caspase-3 and PARP) in SU.86.86 cells increased in a dose-dependent manner. (**B**) SU.86.86 cells were incubated with pegaspargase (4 IU/mL) for various durations, the levels of apoptosis-related proteins exhibited a time-dependent accumulation. *P *values < 0.05 and < 0.01 were denoted by * and **, respectively.

To initially explore the expression of ASNS in SU.86.86 and BxPC-3 cancer cells, K562 cells were used as the control. As shown in Fig. 1A, SU.86.86 and BxPC-3 cell lines exhibited notable deficiency in ASNS, indicating their sensitivity to pegaspargase treatment. Afterwards, pancreatic cancer cells were exposed to different concentrations of pegaspargase for 24, 48, and 72 h. The results of cytotoxicity assay indicated that pegaspargase had an inhibitory effect on cell proliferation, and the inhibitory effect of pegaspargase was dose and time dependent (Fig. 1B). Then, pancreatic cancer cells were exposed to pegaspargase with different concentrations. The results indicated that the apoptosis of SU.86.86 and BxPC-3 cells was significantly increased when they were exposed to pegaspargase. Taking pegaspargase at a concentration of 4 IU/mL as an example, the apoptosis induction rates of SU.86.86 and BxPC-3 cells were 28.7% and 16.9%, respectively. In addition, apoptotic cells in SU.86.86 were mostly in early stage, whereas those in BxPC-3 were mainly in late stage (Fig. 1C and D). To better understand the process of tumor cell death, we further looked into the mechanism of pegaspargase-induced cell apoptosis. In our work, as shown in Figs.2A and B, western blot analysis indicated a dose- and time-dependent elevation in the levels of caspase-3 and PARP cleavage products in SU.86.86 cells, suggesting that pegaspargase-induced apoptosis in pancreatic cancer cells was linked to the activation of caspase-3. These data revealed that pegaspargase elicited prominent cytotoxicity, as well as the increased caspase-mediated apoptosis in pancreatic cancer cells in vitro.

### Autophagy was induced by Pegaspargase in pancreatic cancer cells

**Fig. 3:**
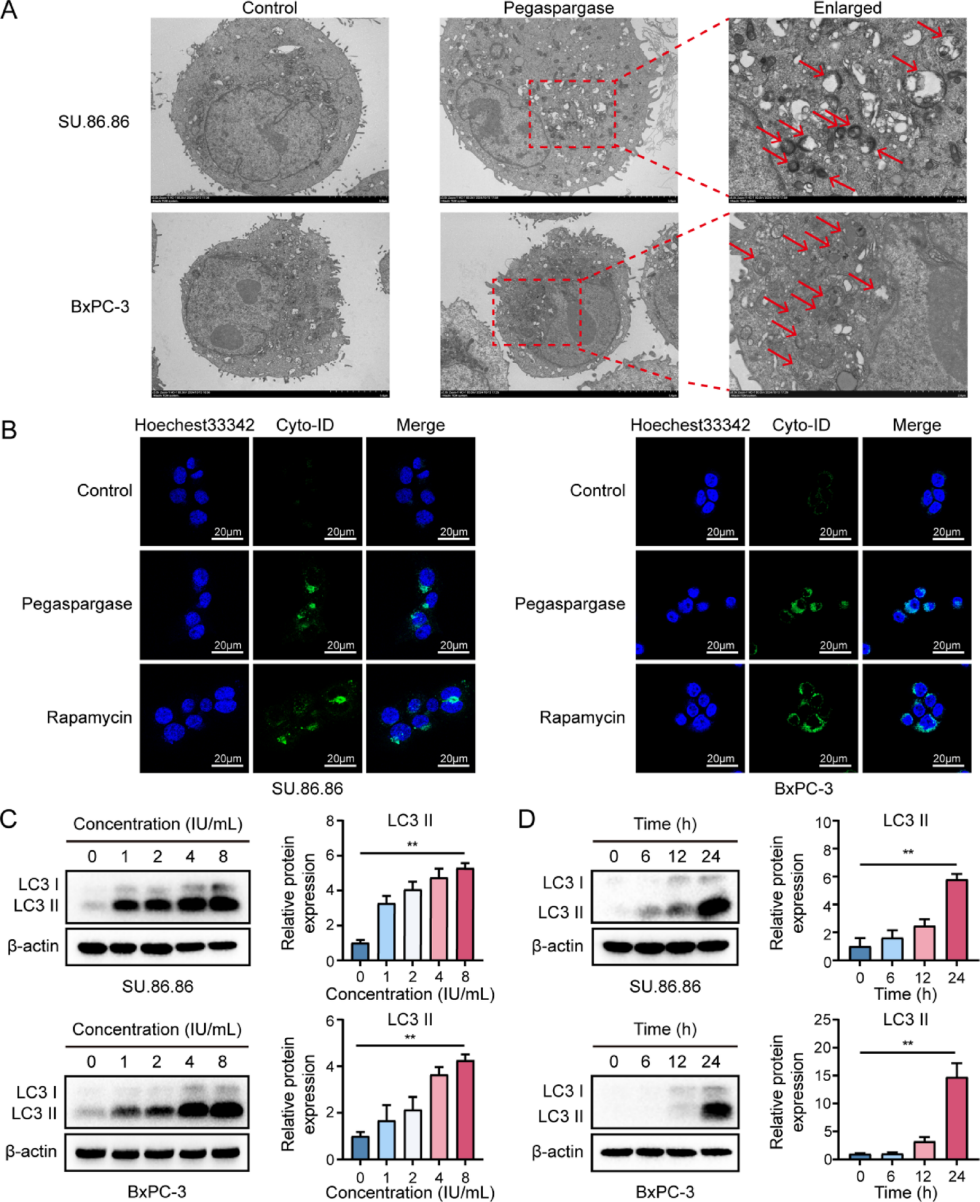
Pegaspargase triggered autophagy in SU.86.86 and BxPC-3 cancer cells. (**A**) The autophagosomes (red arrows) in pegaspargase-treated cancer cells were observed by TEM. (**B**) Pegaspargase-treated cells showed markedly increased green fluorescence intensity by confocal fluorescent microscopy. (**C**, **D**) Autophagy-related protein LC3-II overexpression in both SU.86.86 and BxPC-3 cells. *P *values < 0.05 and < 0.01 were denoted by * and **, respectively

To assess the level of autophagy induced by pegaspargase in pancreatic cancer, we utilized three complementary experimental strategies. As shown in Fig. 3A, autophagosomes (marked with red arrows) in SU.86.86 and BxPC-3 cells were observed by using TEM. Compared with the control group, pegaspargase-treated pancreatic cancer cells exhibited a significant accumulation of autophagosomes. Subsequently, stained tumor cells were imaged using a laser scanning confocal microscope. Here, cells underwent 24-hour exposure to pegaspargase (4 IU/mL), and rapamycin-treated cells were included as a positive control. The results revealed that pegaspargase-treated cells exhibited green fluorescence intensity comparable to the positive control group (Fig. 3B), indicating the accumulation of autophagosomes in pancreatic cancer cells. Lastly, we explored the expression of autophagy-related protein by using the Western bolt. As shown in Fig. 3C, after exposure to pegaspargase for 24 h, SU.86.86 and BxPC-3 cells exhibited overexpression of LC3 II, with its expression level rising as pegaspargase concentrations increased. Similarly, when cells were exposed to pegaspargase (4 IU/mL), the results showed that the expression of LC3 II was significantly increased with the increase of exposure time of pegaspargase (Fig. 3D). These data strongly suggested that pegaspargase promoted SU.86.86 and BxPC-3 cells to autophagy.

### The Akt/mTOR signaling pathway regulated autophagy in response to Pegaspargase treatment

**Fig. 4:**
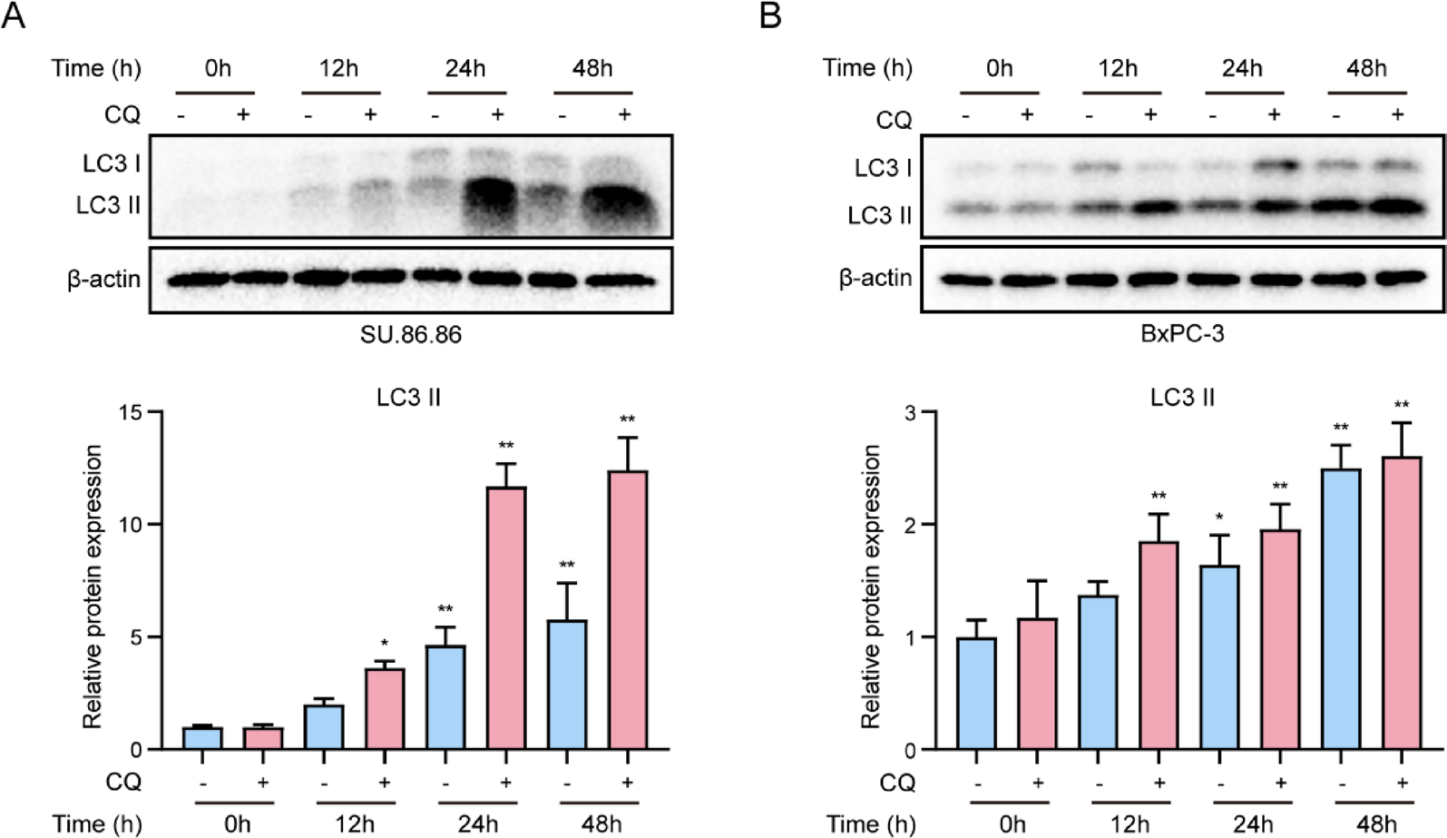
Pegaspargase activated autophagic flux in pancreatic cancer cells. (**A**, **B**) SU.86.86 and BxPC-3 cells were incubated with pegaspargase (4 IU/mL) for various durations, the autophagic flux in pegaspargase-treated cells was analyzed by western blot analysis. *P *values < 0.05 and < 0.01 were denoted by * and **, respectively

**Fig. 5:**
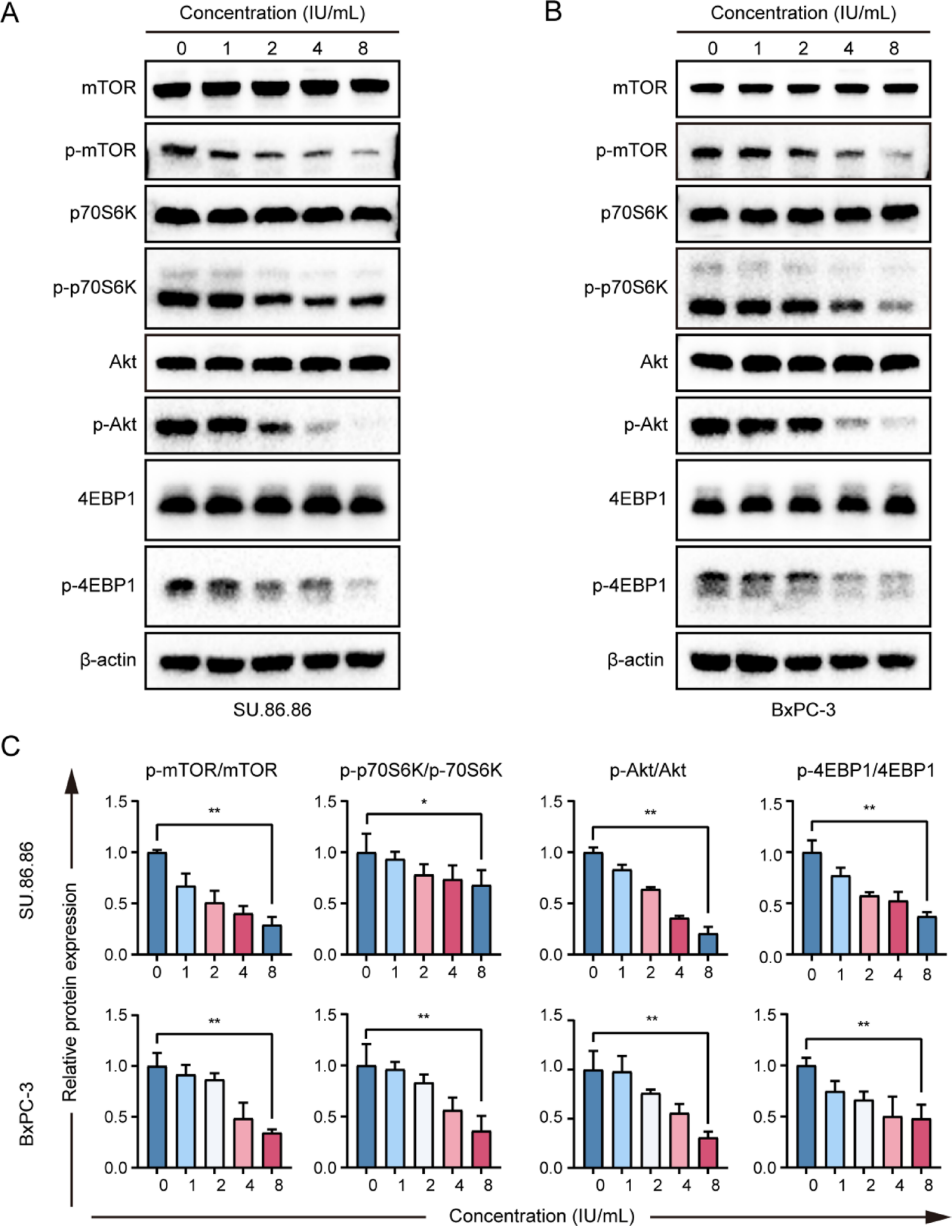
Autophagy was regulated by the Akt/mTOR signaling pathway in pegaspargase-treated pancreatic cancer cells (**A**, **B**) Western blot analysis of phosphorylated and total protein levels of Akt, mTOR, p70S6K, and 4EBP1 in SU.86.86 and BxPC-3 cells treated with indicated concentrations of pegaspargase for 24 hours. (**C**) Bar graphs depict the quantified ratios of p-protein/total protein. *P* values < 0.05 and < 0.01 were denoted by * and **, respectively

To gain a deeper understanding of the dynamic changes in the process of autophagy, we evaluated the autophagic flux in pegaspargase-treated pancreatic cancer cells. By introducing CQ (5 µM), an inhibitor of autophagosome-lysosome fusion that suppresses autophagic flux, a significant positive correlation was observed between the level of LC3-II and the duration of pegaspargase treatment. In addition, when cotreated with CQ, the expression of LC3-II was further upregulated (Figs. 4A and B), indicating that pegaspargase induced a significant autophagic flux in pancreatic cancer cells. In order to explore the molecular mechanisms, we further investigated the autophagy-related signaling pathways. In this study, SU.86.86 and BxPC-3 cells were treated with different concentrations of pegaspargase for 24 h. Western blot analysis revealed that the phosphorylation levels of mTOR and its upstream activator, Akt, were negatively correlated with the concentration of pegaspargase. Quantitative analysis revealed that the ratios of p-mTOR/mTOR, p-Akt/Akt, p-p70S6K/p70S6K, and p-4EBP1/4EBP1 were significantly reduced upon pegaspargase treatment in a concentration-dependent fashion (Fig. 5A and BC). These results implied that pegaspargase-triggered autophagy in SU.86.86 and BxPC-3 cells was mediated through suppression of the Akt/mTOR signaling pathway.

### Autophagy inhibitor accelerated the pegaspargase-induced apoptosis in SU.86.86 and BxPC-3 cells

**Fig. 6:**
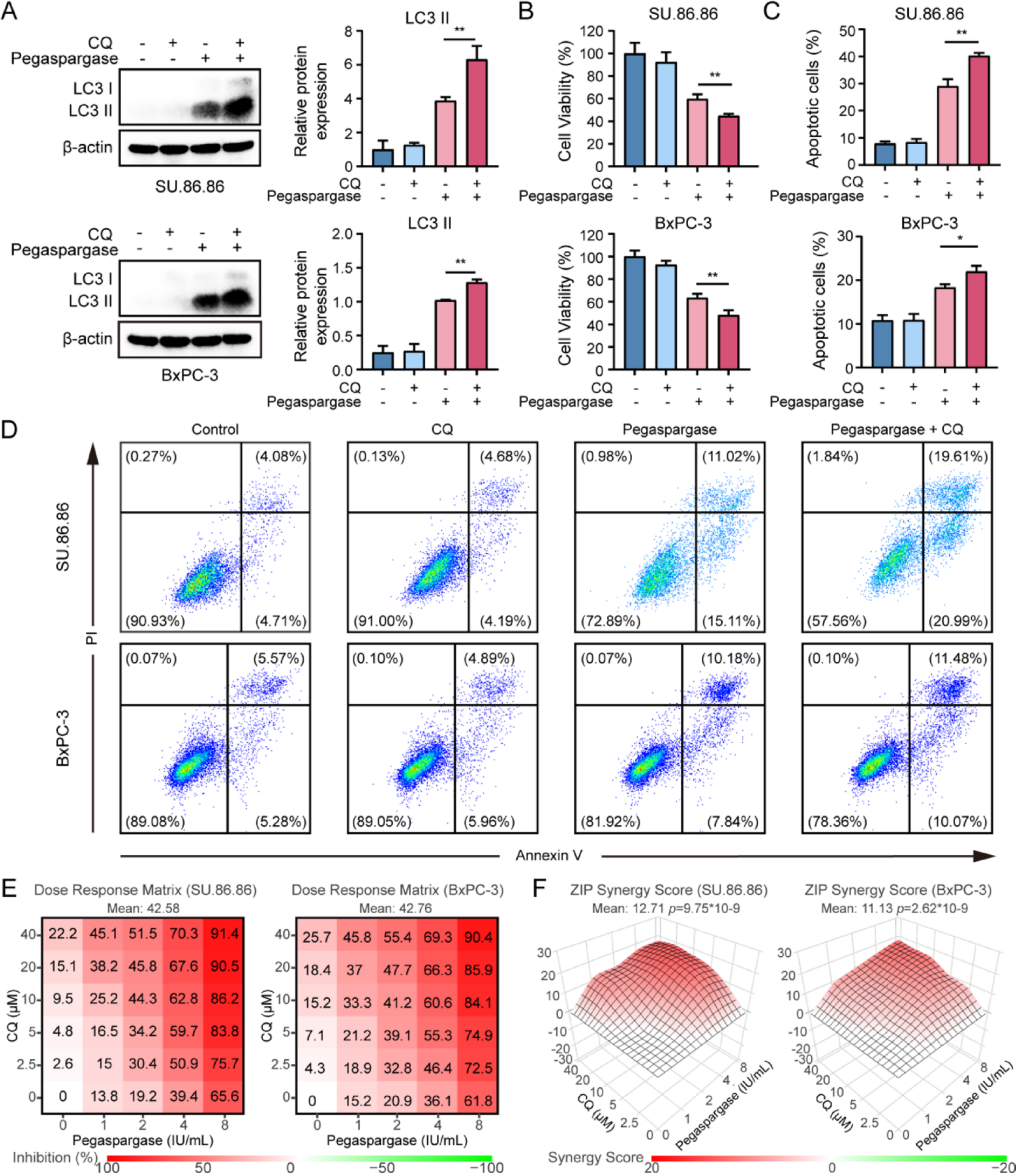
Autophagy inhibitor enhances the apoptosis of pancreatic cancer cells following treatment with pegaspargase (**A**) The expression of LC3 in pancreatic cancer cells was measured after introducing CQ. (**B**) The viability of cells exposed to pegaspargase and/or CQ for 48 hours was assessed. (**C**, **D**) The apoptotic rate of pancreatic cells under different treatment modalities was analyzed via flow cytometry. (**E**) Dose-response matrices of pegaspargase and CQ in SU.86.86 and BxPC-3 cells. (**F**) Drug synergy was analyzed in pancreatic cancer cells using the ZIP model. *P *values < 0.05 and < 0.01 were denoted by * and **, respectively.

Subsequently, we set out to investigate the specific function of pegaspargase-induced autophagy in pancreatic cancer cells. As shown in Fig. 6A, pegaspargase (4 IU/mL) and/or CQ (5 µM) were applied to cells for 24 h. The results showed that CQ successfully inhibited pegaspargase-triggered autophagy in both SU.86.86 and BxPC-3 cells, as evidenced by the increase in LC3 II levels when pegaspargase was combined with CQ. Upon treating cells with pegaspargase and/or CQ for 48 h, cell viability assays demonstrated a remarkable decrease in cell viability within the group receiving combination treatment (Fig. 6B), indicating that cotreatment with CQ increased the sensitivity of pancreatic cancer cells to pegaspargase. Furthermore, after 48-hour exposure to pegaspargase, flow cytometry results (Figs. 6 C and D) showed that, compared to pegaspargase (4 IU/mL) treatment alone, the proportion of apoptotic cells in SU.86.86 and BxPC-3 cells was considerably raised by the combination of CQ. Specifically, in the combined treatment group, the apoptosis rate of SU.86.86 cells increased to 39.9%, while that of BxPC-3 cells rose to 21.9%. Figure 6E showed the dose-response matrix of pegaspargase and CQ in SU.86.86 and BxPC-3 pancreatic cancer cells. The ZIP model confirmed strong synergistic effects (synergy score > 10) between pegaspargase and chloroquine across multiple concentration combinations in pancreatic cancer cells (Fig. 6F). Overall, these observations suggested that autophagy protected pancreatic cancer cells treated with pegaspargase. Autophagy suppression may enhance the cytotoxicity of pegaspargase.

### Autophagy suppression potentiated the antitumor activity of Pegaspargase in vivo

**Fig. 7:**
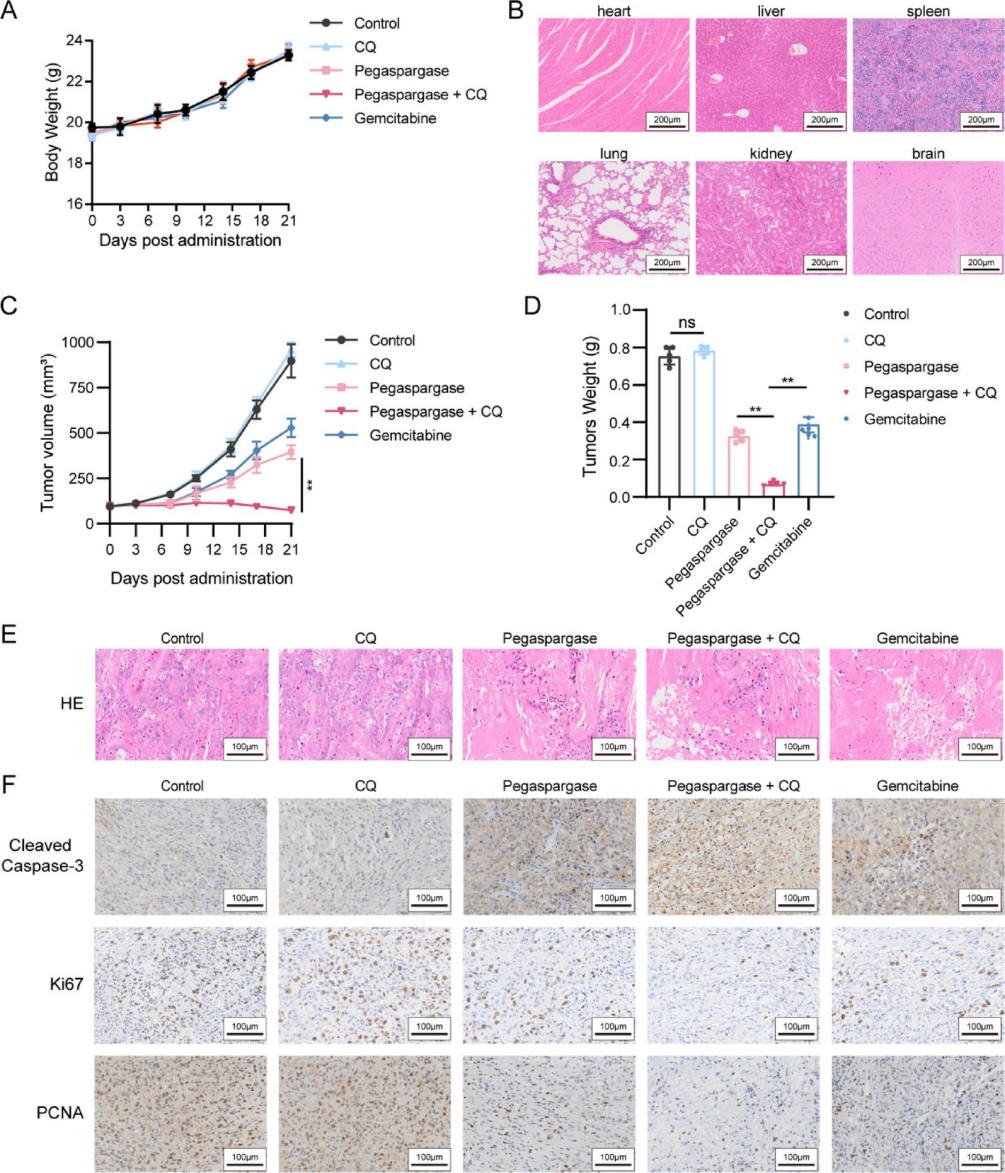
Suppressing autophagy enhances pegaspargase's antitumor in vivo (**A**) Mouse body weight changes during the treatment period. (**B**) Histopathological examination of vital organs (heart, liver, spleen, lung, kidney, and brain). (**C**) Tumor volumes of different groups were measured and recorded regularly. (**D**) Tumor weights of all groups were recorded after treatment cessation. (**E**) The histopathological changes in tumor tissues under different drug administration regimens were exhibited through Hematoxylin and eosin staining. (**F**) Immunohistochemical staining for cleaved caspase-3, Ki-67 and PCNA of tumor tissues from the indicated treatment groups.

To investigate whether inhibiting autophagy can improve pegaspargase’s antitumor activity in vivo, a xenograft tumor model was set up using the SU.86.86 cell line. When the subcutaneous tumor reached a volume of approximately 100 mm³, the mice were allocated into five distinct groups at random: negative control group, pegaspargase group, CQ group, combined CQ and pegaspargase group, and gemcitabine-positive control group. No significant reduction in body weight was observed in any of the groups. None of the treatment groups exhibited a significant reduction in body weight (Fig. 7A). Behavioral monitoring during treatment revealed no evidence of systemic toxicity (e.g., lethargy, motor impairment) or gastrointestinal adverse effects (e.g., diarrhea). Following the treatment period, histopathological examination of major organs, including the heart, liver, spleen, lung, kidney, and brain, revealed no significant pathological alterations (Fig. 7B). As shown in Fig. 7C and D, pegaspargase (1000 IU/kg) alone dramatically decreased the growth rate and size of tumors in mice as compared to the negative control group. Notably, the slowest tumor growth rate was observed in the combined treatment group (50 mg/kg). Histological analysis of the tumors from different groups revealed that the tumor cells count in the pegaspargase-treated groups significantly decreased, especially in the combination group of pegaspargase and CQ. Specifically, as shown in Fig. 7E, the tumor cells in the combined treatment group exhibited a decline in the overall density of tumor cells. The immunohistochemistry results demonstrated that, compared with the pegaspargase monotherapy group, the combination therapy group showed significantly upregulated expression of cleaved caspase-3 and markedly downregulated expression of Ki-67 and PCNA (Fig. 7F). In conclusion, these results demonstrated that autophagy suppression exhibited synergistic antitumor efficacy with pegaspargase against pancreatic cancer in vivo, consistent with our previous in vitro experimental findings.

(A) Mouse body weight changes during the treatment period. (B) Histopathological examination of vital organs (heart, liver, spleen, lung, kidney, and brain). (C) Tumor volumes of different groups were measured and recorded regularly. (D) Tumor weights of all groups were recorded after treatment cessation. (E) The histopathological changes in tumor tissues under different drug administration regimens were exhibited through Hematoxylin and eosin staining. (F) Immunohistochemical staining for cleaved caspase-3, Ki-67 and PCNA of tumor tissues from the indicated treatment groups.

## Discussion

The metabolic distinctions between healthy and tumor cells are a pivotal basis for exploring antitumor drug strategies, and amino acid depletion is recognized as a means to inhibit tumor progression [29, 30]. For example, cysteinase showed potential for cancer treatment due to its ability to reduce the bioavailability of cysteine [31]. In vivo, targeting arginase exhibited obvious antiproliferative effects against various cancer cell lines [32]. In addition, asparaginase leveraged the characteristic asparagine metabolism addiction of ALL due to the lack of ASNS to exert its anticancer effects [33]. Recently, it has been reported that asparaginase also showed ideal therapeutic effects in other hematological malignancies, while its effect on solid tumors is not so ideal [7, 34]. To develop a predictive model, we chose pancreatic cancer for the following reasons: Firstly, pancreatic cancer treatment still has a relatively slow development [35]. Secondly, about half of pancreatic cancer patients exhibit absent or low expression of ASNS, which offers an opportunity for asparaginase’s clinical application in treating pancreatic cancer. Indeed, Dufour E et al. also demonstrated that certain pancreatic cancer cell lines exhibited sensitivity to asparaginase treatment both in vitro and in vivo [36]. However, its cytotoxic mechanism is still unknown. In our work, we initially screened SU.86.86 and BxPC-3 pancreatic cancer cell lines, which exhibited low expression of ASNS. We used pegaspargase, which is a relatively safe and stable asparaginase formulation, to deplete asparagine. The results showed that pegaspargase significantly inhibited the growth of pancreatic cancer cells in vitro, which was in line with previous reports. Then, we observed that pegaspargase induced caspase-related apoptosis, further elucidating the cytotoxic mechanism of pegaspargase on pancreatic cancer cells.

The main reason limiting the extensive application of asparaginase in solid tumors is the specific resistance of solid tumor cells to asparaginase, as well as the dose-limiting toxicity of asparaginase [37, 38]. In response to these challenges, previous studies have proposed a series of potentially viable strategies. For instance, Nakamura A et al. generated GCN2 inhibitor and demonstrated that it could enhance tumor cell sensitivity to asparaginase by suppressing ASNS levels [39]. Sun J et al. revealed that tumor cells resist asparaginase action by promoting amino acid metabolism through SLC1A3 expression, and the blockade of SLC1A3 improved the efficacy of asparaginase in solid tumors [40]. Furthermore, asparaginase therapy activated autophagy in laryngeal squamous cell carcinoma, as well as lung adenocarcinoma. When combined with autophagy inhibitors, a significant enhancement in tumor response to asparaginase was observed [27, 41]. Indeed, a Phase IIb clinical study (NCT02195180) conducted by Hammel P and colleagues revealed that he combination of eryaspase and chemotherapy could prolong the lifespan of patients with advanced pancreatic cancer [42]. Regrettably, the subsequent Phase III study (NCT03665441) by the same team failed to improve the OS of pancreatic cancer patients treated with eryaspase in combination with chemotherapy [43]. Here, we reported for the first time the induction of autophagy and activation of autophagic flux in SU.86.86 and BxPC-3 pancreatic cancer cells following exposure to pegaspargase. In addition, this autophagic response was effectively inhibited by the use of autophagy inhibitors in vitro. Mechanistically, we found that pegaspargase inhibited the Akt/mTOR signaling pathway, which subsequently induced. These findings align with the established understanding that autophagy-related pathways involve multiple key regulatory factors [44, 45].

Autophagy is involved in many physiological and pathological processes [46, 47]. When cells encounter malnutrition or stress, autophagy can be engaged by tumor cells as a survival strategy to escape programmed cell death [48]. Previous studies have reported that inhibition of autophagy could result in the death of nutrient-starved cells [25, 26]. Given that autophagy may provide energy and raw materials for cells through self-degradation [49], targeting autophagy has attracted increasing attention in oncology research as a novel therapy. In this study, we found that inhibition of autophagy not only enhanced the cytotoxicity of pegaspargase and induced significantly higher apoptosis rate. Quantitative synergy analysis showed a significant synergistic effect between pegaspargase and chloroquine in both SU.86.86 and BxPC-3 pancreatic cancer cell lines. Further experiments proved the efficacy of pegaspargase in vivo, and the application of CQ significantly enhanced the ability of pegaspargase to limit the growth of pancreatic cancer cell. Based on above findings, we hypothesized that this novel therapy might enhance the effect of pegaspargase on pancreatic cancer and weaken its drug resistance partially.

In summary, pegaspargase is a classic therapy for hematological cancers recently, however, the application effect of pegaspargase on solid tumors is greatly limited.

In this study, we found that the caspase-dependent apoptosis was markedly enhanced by pegaspargase in vitro in SU.86.86 and BxPC-3 cancer cells. Inhibition of the Akt/mTOR signaling pathway by pegaspargase might induce the accumulation of autophagy. Importantly, the effectiveness of pancreatic cancer was greatly enhanced by combination with CQ both in vitro and in vivo. Our study emphasized that inhibiting autophagy could enhance the sensitivity of pancreatic cancer to pegaspargase. Beyond chemical inhibition of autophagy, we speculated that other approaches to suppressing autophagy, such as modulating autophagy-related signaling pathways and targeting autophagy-related genes, could also address resistance to pegaspargase. The combination of pegaspargase with autophagy inhibition might represent a viable novel treatment approach for pancreatic cancer in the future.

## Data Availability

Upon receipt of a reasonable request, the author will provide all datasets involved in this study.
